# Acute Type A Aortic Dissection in a 36-Week Pregnant Patient

**DOI:** 10.1155/2013/390670

**Published:** 2013-05-28

**Authors:** Erol Kohli, Sharhabeel Jwayyed, Gary Giorgio, Mary Colleen Bhalla

**Affiliations:** Department of Emergency Medicine, Summa Akron City Hospital, 525 East Market Street, Akron, OH 44309, USA

## Abstract

Aortic dissection is a relatively rare yet often fatal condition. Early recognition and treatment are crucial for survival. While the majority of patients who present with aortic dissection are older than 50 years of age and have a history of hypertension, younger patients with connective tissue disease, bicuspid aortic valves, and a family history of aortic dissection are also at an increased risk for developing this condition. A review of the literature revealed a paucity of published cases describing the successful, emergent repair of acute type A aortic dissections in third- trimester gravid patients. We present the case of the successful diagnosis and surgical repair of a 41-year-old female who presented to the emergency department with an acute type A aortic dissection at 36 weeks of gestation.

## 1. Introduction

Aortic dissection is a rare complication of pregnancy with significant morbidity and mortality for both the mother and infant. In the general population, aortic dissection has an estimated incidence of 2.9 per 100,000 person years [1]. In pregnancy, acute type A aortic dissection has an overall incidence of 0.4 cases per 100,000 person years [2]. Common risk factors for developing aortic dissection include hypertension, collagen disorders including Marfan/Ehlers-Danlos syndromes, bicuspid aortic valve, positive family history, trauma, and cocaine use [3–7]. Mechanisms leading to aortic dissection involve a weakening of the layers of the aorta allowing blood to enter the media and separate the intimal and adventitial layers creating a false lumen. Acute aortic dissection during the third trimester of pregnancy has been attributed to hemodynamic alterations that occur in late pregnancy. These changes include increased total circulatory volume, increased systemic blood pressure, and structural changes in the aortic wall secondary to the hormonal effects of estrogen and progesterone [4]. A high index of suspicion by the emergency physician in addition to successful coordination of care between the emergency physician, obstetrician, and cardiothoracic/vascular surgeon is necessary for a successful outcome.

## 2. Case Report

A previously healthy 41-year-old G1P0 female presented to the emergency department during the 36th week of pregnancy with a sudden onset of severe midsternal chest pain radiating to her back. The pain began roughly 30 minutes prior to her presentation to the ED and occurred as she was getting out of the swimming pool. She described the pain as continuous, 10/10 in severity, worsened with movement and breathing, and relieved by nothing. Associated symptoms include nausea, vomiting, diaphoresis, and light-headedness. She denied contractions, vaginal bleeding, or leakage of fluid. The patient denied any significant past medical history other than mild hypertension that developed during the third trimester of her pregnancy. Her only medication was prenatal vitamins and she denied any alcohol, tobacco, or drug use. The patient had no known risk factors and a negative family history of aortic dissection or connective tissue disease. 

### 2.1. Physical Examination

Physical examination revealed an overweight female appearing her stated age of 41 years in moderate distress secondary to chest pain and nausea. On initial presentation, her BP was 94/30 mmHg, heart rate 71 beats/min, respiratory rate 16 breaths/min, oxygen saturation 99% on room air, and temperature of 95.3°F (35.1°C). Her neck was supple without jugular venous distension. Cardiovascular exam revealed regular rate and rhythm with no murmurs, rubs, or gallops. Lungs were clear to auscultation bilaterally without wheezes, rhonchi, or rales. Abdomen was gravid, consistent with gestational age. Fetal heart tones were 155. The exam of her extremities showed 1+ right radial pulse and 2+ left radial pulse. She had no palpable pedal pulses and bilateral 1+ edema of her lower extremities. The patient was anxious but cooperative with no focal neurologic deficits.

### 2.2. Diagnostic Tests and Clinical Course

**Figure 1:**
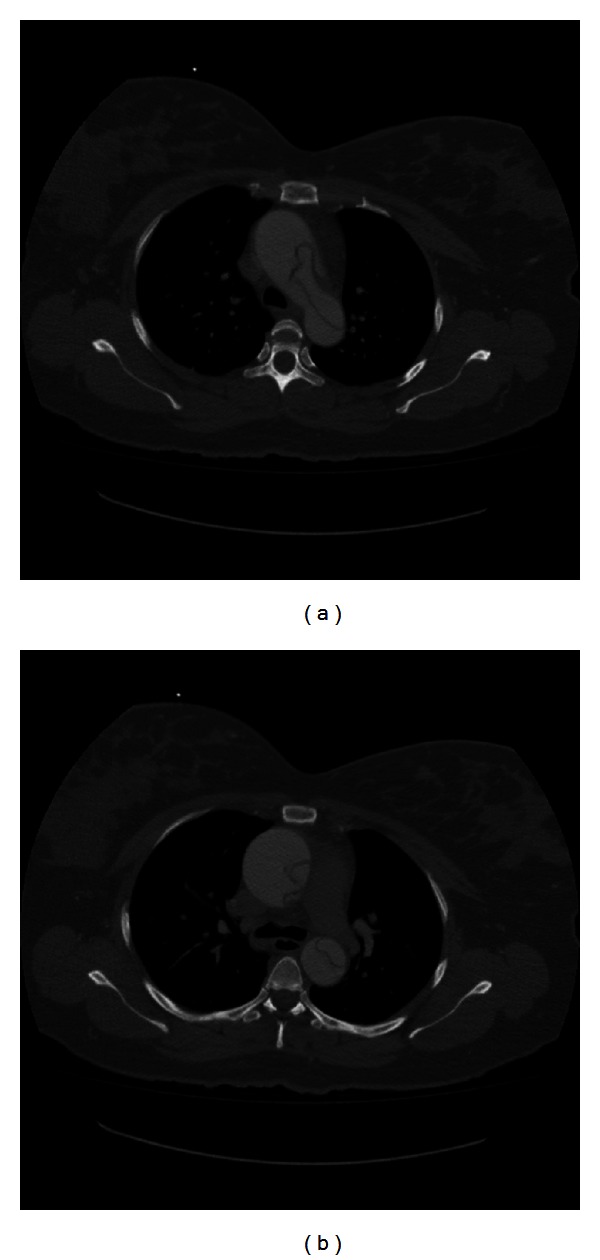
Axial view demonstrating acute dissection in ascending and descending aorta.

**Figure 2:**
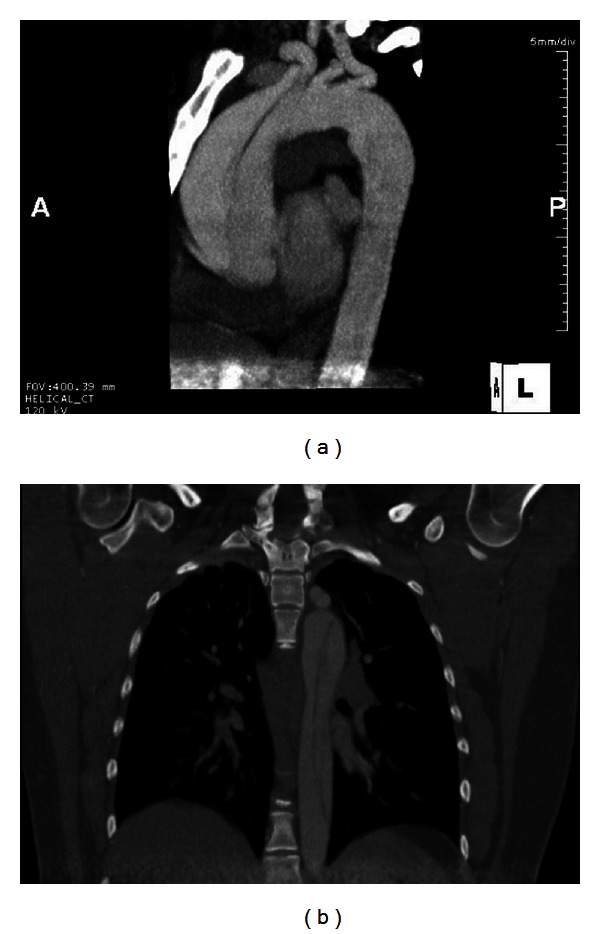
Sagittal and coronal views demonstrating acute dissection in ascending and descending aorta.

**Figure 3:**
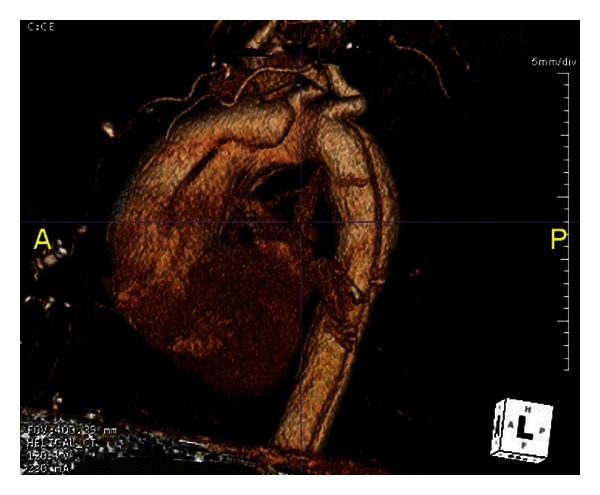
CTA reconstruction demonstrating acute dissection in ascending and descending aorta.

EKG showed a sinus rhythm with a rate of 75 and no signs of ischemic changes. Labs revealed an unremarkable BMP, coagulation study, and CBC with a hemoglobin of 10.8 and hematocrit of 32.2. We obtained a CT angiography of the chest due to our high index of suspicion for an acute aortic dissection. CT revealed a Stanford type A aortic dissection involving the ascending and descending aorta. The dissection started at the aortic root and extended inferiorly into the descending aorta and upper abdomen. The true lumen was found to be on the left side near the aortic root and included the origins of the great vessels and extended inferiorly along the anterior and left sides of the descending aorta (Figures 1, 2, and 3). Upon reviewing the CT, we immediately notified the obstetric and vascular teams and the patient was taken to the operating room for an emergent cesarean section and repair of her aortic dissection. Followup at five weeks revealed a healthy mother and infant.

## 3. Discussion

Aortic dissection is a rare complication of pregnancy with significant morbidity and mortality for both the mother and infant. Our case report highlights the early, successful diagnosis and surgical repair of a healthy 41-year-old female who presented with an acute type A aortic dissection in the third trimester of pregnancy. A recent 2011 population-based study of acute type A aortic dissection in pregnancy revealed an overall incidence of 0.4 cases per 100,000 person-years and a prehospital mortality rate of 53% [2]. The mortality rate for untreated proximal aortic dissections increases by 1–3% per hour after presentation and is roughly 25% during the first 24 hours and up to 70% at 1 week and 80% at 2 weeks [5]. A longitudinal study over a period of 27 years discovered that aortic dissection was the initial clinical diagnosis in only 15% of patients presenting with an aortic dissection [1]. In general, Stanford type A dissections involve the ascending aorta and are managed surgically while type B dissections involved the descending aorta and are managed medically. While there are no firmly established practice guidelines, it has been suggested that before 28 weeks of gestation, aortic repair should proceed without cesarean section and after 32 weeks gestation, emergency cesarean section precede aortic repair [6]. Between 28 and 32 weeks, the risks and benefits of early delivery should be considered.

Although relatively rare, clinicians must maintain a high index of suspicion for aortic dissection in any pregnant female who presents with acute onset chest pain. Early recognition by the emergency physician in addition to successful coordination of care between the emergency physician, obstetrician, and cardiothoracic/vascular surgeon is necessary for a successful outcome.
